# Does type of hospital ownership influence physicians' daily work schedules? An observational real-time study in German hospital departments

**DOI:** 10.1186/1478-4491-7-41

**Published:** 2009-05-27

**Authors:** Stefanie Mache, Cristian Scutaru, Karin Vitzthum, David Quarcoo, Norman Schöffel, Tobias Welte, Burghard F Klapp, David A Groneberg

**Affiliations:** 1 Institute of Occupational Medicine, Charité – School of Medicine, Free University and Humboldt University, Berlin, Germany; 2 Department of Respiratory Medicine, Hannover Medical School, Hannover, Germany; 3 Department of Medicine/Psychosomatics, Charité – School of Medicine, Free University and Humboldt University, Berlin, Germany

## Abstract

**Background:**

During the last two decades the German hospital sector has been engaged in a constant process of transformation. One obvious sign of this is the growing amount of hospital privatization. To date, most research studies have focused on the effects of privatization regarding financial outcomes and quality of care, leaving important organizational issues unexplored. Yet little attention has been devoted to the effects of privatization on physicians' working routines. The aim of this observational real-time study is to deliver exact data about physicians' work at hospitals of different ownership. By analysing working hours, further impacts of hospital privatization can be assessed and areas of improvement identified.

**Methods:**

Observations were made by shadowing 100 physicians working in private, for-profit or non-profit as well as public hospital departments individually during whole weekday shifts in urban German settings. A total of 300 days of observations were conducted. All working activities were recorded, accurate to the second, by using a mobile personal computer.

**Results:**

Results have shown significant differences in physicians' working activities, depending on hospital ownership, concerning working hours and time spent on direct and indirect patient care.

**Conclusion:**

This is the first real-time analysis on differences in work activities depending on hospital ownership. The study provides an objective insight into physicians' daily work routines at hospitals of different ownership, with additional information on effects of hospital privatization.

## Background

Since the Second World War the German health system has been detached from the general rules of commercial necessity [1]; this was about to change, beginning in 1990. Nowadays an increasing economic efficiency of German hospitals is the driving engine when it comes to decision-making in the medical sector [2,3]. A steep increase in health care costs has caused an additional financial burden to the German health care system [4]. In addition, hospitals must compensate for declining public financial resources [5].

For these reasons, reforms have been adopted in recent years, causing restrictions on the funding situation and an initial increase in competition among health services providers [6,7]. Objectives such as effectiveness, appropriateness, quality and cost-effectiveness as well as patient involvement gained an increasing importance and shaped the behaviour of health care providers and payers [5].

At present hospitals compensate for declining financial resources by reducing their personnel expenditures, increasing the patient load per physician and redesigning medical working shifts [7]. As a consequence, medical services have become more formalized, physicians are expected to work overtime and activities involving direct patient contact are in danger of diminishing in the face of economic realities [7,8]. This paper discusses these issues with regard to hospital privatization [9-11], which has now become a popular strategy in the German health care system in an attempt to make hospitals more profitable [12].

Currently the German hospital situation is characterized by the simultaneous existence of various types of ownership. Following the definition of the Statistical Offices of the federal states, there are three hospital types in Germany: (1) public hospitals run by the local authorities, the city, communities and the "Länder"; (2) private hospitals run as free commercial enterprises; and (3) voluntary non-profit hospitals run by non-profit organizations such as churches or non-profit-making organizations, such as the German Red Cross [13].

In 2005, the number of private, for-profit hospitals increased by 7.4% compared to previous years, bringing the total share up to 44.2%. At the same time, the number of public hospitals decreased, from 46.0% to 35.1% [14]. The fraction of non-profit hospitals has remained relatively constant over the same period [13].

Most comparative research has focused mainly on differences between hospital types regarding costs, quality of care and patients' satisfaction, leaving other organizational issues unexplored. Despite its importance, little attention has been devoted to the effects on physicians' work at hospitals of different ownership. Only limited subjective reporting on questionnaires provided information on this research focus, concerning higher burnout levels and workloads at private hospitals [15].

Unfortunately, objective data on physicians' work activities in hospitals of different ownership types is missing. By analysing working routines, areas of differences between ownership types can be assessed more precisely and further impacts of hospital privatization can be identified. To prove potential differentiations, we conducted a real-time, objective monitoring study to deliver exact data about physicians' work in hospitals of different ownership. The long-term aim of the study is to provide suggestions to improve working conditions in German health care services.

## Methods

### Participants and setting

The study was conducted at 12 urban hospitals, all situated in or around Berlin, Germany. Hospitals were grouped into three main ownership types: (1) public hospitals run by the local authorities, the towns and the "Länder"; (2) private, voluntary, non-profit-making hospitals run by churches or non-profit-making organisations; (3) private, for-profit hospitals run as free commercial enterprises.

The hospitals were chosen because of their similarities in size (number of inpatient beds) and specific care profile. Based on information of the German Federal Office of Statistics, they were also comparable to other German hospitals of the same ownership type [16]. The participating hospitals specialized in at least one of the following medical care specialties: paediatrics, cardiology, haematology and oncology, respiratory medicine and neurology.

Table 1 represents a comparison between differently owned hospitals regarding the average number of beds, physicians and nurses working at a hospital department. Data based on calculations of total values of all included hospital departments.

**Table 1:** Descriptive statistics of hospital department characteristics: comparison between hospital ownership types

Variable	Private, for-profit hospital department		Public hospital department		Private. non-profit hospital department		chi^2^
	Mean	S.D.	Mean	S.D.	Mean	S.D.	

Number of patient beds	16.33	1.75	16.66	3.26	17.00	3.00	0.188

Number of doctors	14.17	3.19	15.83	2.78	15.67	3.05	1.061

Number of nurses	35.67	9.43	35.83	8.89	30.33	4.72	1.185

All junior physicians working in the chosen hospitals were invited by a written request to participate in the study. After the study obtained the institutional review board's approval, a sample of 100 physicians volunteered to take part. The mean age in the sample was 32 years (SD = 3.7); the average time working as a physician was three years (SD = 2.36). No significant differences were found among the participants of the three ownership types regarding their age or working experience. All physicians included in the study are full-time employees.

### Procedure

The current study was an observational field investigation employing the shadowing method. The data collection began on 1 October 2007 and ended on 1 December 2008. In total, 300 working days were recorded (20 observation periods in each hospital ownership type (n = 3) per medical specialty (n = 5) to ensure an equal distribution). Table 2 represents the uniform distribution of observation days per medical specialty.

**Table 2:** Number of observation days per medical specialty

Variable	Frequency
Medical specialty	

• Paediatrics	60

• Respiratory medicine	60

• Haematology and oncology	60

• Cardiology	60

• Neurology	60

Total	300

In shadowing, a researcher observes a physician unobtrusively and takes notes of each point in time that a physician starts a new job task. A specially designed computer program, inserted in an Ultra Mobile PC (Samsung Q1, Samsung Electronics GmbH, Schwalbach, Germany), was used to record each job task in real-time, accurate to the second [17].

Eleven task categories were determined to represent the major job tasks undertaken by physicians during their typical work shifts (Table 3). In addition, the number of patients in treatment was recorded during the investigation period.

**Table 3:** Categorization of job tasks

Task name	Description
Internal communication/Meetings	Conversation with physicians or other medical staff; advanced training

Documentation and Administrative tasks	Writing discharge letters, administrative work, daily notes, disability letters

Ward round/Admission to hospital	Examination in the sickbed by one or several doctors; obtaining patient history and examining patients when they enter the hospital

Indirect patient care	Chart rounds, literature research, charging infusion plans, evaluation of findings

Direct patient care	Clinical examinations, scientifically documented tests

Communication with patients	Face-to-face communication with the patient, family meetings

Resting period: "breaks"	Time of recovery (e.g. lunch), bathroom breaks

Walking around	Walking around between tasks

Work obstacles	Searching for documents, waiting for patients, reports, computer problems

Teaching	Activities of educating medical students

Miscellaneous	Time spent on personal activities (e.g. changing working clothes)

The research assistant recorded all work activities throughout complete daily shifts. Daily shifts began at the time the doctor arrived at the hospital ward and ended when he or she left the hospital. This constituted one observation period. To diminish the possibility of affecting behaviour by the physician's awareness of participating in a research study, the data collector stood at least three meters from the physician and was informed not to initiate conversation with him or her.

### Validity of the task classification

The first step was to create a list of task categories performed by all physicians regardless of the medical specialty. All physicians verified the categories for correctness. Afterwards, observations in each medical field and hospital department took place to prove the content validity. These observations lasted three working shifts in each hospital department.

### Inter-observer reliability

Two researchers tested the methodology by collecting data simultaneously but independently. The main investigation did not start until an inter-observer agreement of 85% was recorded in each medical field.

### Data analysis

All working events were documented in real time and entered into an Excel database (Microsoft Cooperation^®^) for analysis. In addition to descriptive statistics, (non-parametric) variance analyses were conducted to examine whether there were significant time differences in performing work activities between hospital ownership types.

The included data was not normally distributed, which contradicted the assumptions of using ANOVA – the parametric choice for comparisons of means between three groups or more [18]. Therefore, the non-parametric alternative, the Kruskal-Wallis test, was used for the data analysis to compare the three independent groups. In addition, a correlation analysis was conducted by calculating Spearman's rank correlation coefficients.

A p-value of less than .05 was identified as a significant result. Values were given as mean and standard deviations (SD). Statistical analysis was conducted using the SPSS^® ^Software Package for Social Sciences; Version 17.0. All data were kept anonymous and confidential.

## Results

In aggregate, 2780 hours of work activity were recorded during the study period. Additional file 1 presents the results of all work activities undertaken by the physicians during the investigation time.

### Differences in length of workday depending on ownership

Results of the univariate analysis showed a significant difference regarding working schedule. Physicians in public hospitals worked significantly longer hours than physicians working in private hospitals (chi^2 ^= 38.52, df = 2, p < .001).

The average working time per shift at a private hospital was 8:52:52 hours (CI 95% = 8: 40:42 h to 9:05:02 h), in contrast to 09:48:21 hours at public hospitals (CI 95% = 9:35:10 h to 10:01:32 h) and 09:06:56 hours at non-profit hospitals (CI 95% = 8:55:27 h to 9:18:25 h).

During a shift, an average of 36 minutes was spent on rest periods in private, for-profit hospitals (CI 95% = 0:32:22 h to 0:39:42 h), 22 minutes in public hospitals (CI 95% = 0:19:47 h to 0:25:21 h) and 27 minutes in private, non-profit hospitals (CI 95% = 0:24:21 h to 0:30:44 h) (chi^2 ^= 28.26, df = 2, p < .001).

Meetings, documentation tasks and indirect patient care scored highest per observational period in all hospitals (see Additional file 1).

### Differences in meetings and internal communication depending on ownership

Time wise, the major part of a single working day was spent on meetings and internal communication, regardless of type of ownership. Moreover, no significant difference was found between types of ownership chi^2 ^= 1.588, df = 2, p = .452).

### Differences in administrative and documentation tasks depending on ownership

Across all shifts, physicians of public hospitals spent significantly more time on documentation and administrative tasks (M = 1:52:00 h, CI 95% = 1:42:48 h to 2:01:12 h), compared to physicians of private, for-profit hospitals (M = 1:44:27 h, CI 95% = 1:35:45 h to 1:53:09 h) and private, non-profit hospitals (M = 1:31:56 h, CI 95% = 1:23:32 h to 1:40:20 h) (chi^2 ^= 7.87, df = 2, p < .05). In addition, a significant positive correlation was found between documentation tasks and general working hours (r = .14, p < .05).

### Differences in indirect patient care

Another large time commitment was allotted for indirect patient care. Overall, physicians of private hospitals spent significant less time on indirect patient care, including, for instance, chart rounds, requesting medical reports, literature research or changing infusion plans, than physicians of other types of hospitals (chi^2 ^= 16.95, df = 2, p < .001).

### Differences in time for ward rounds and direct patient care

A physician working at a private, for-profit hospital spent 1:15:25 hour on ward rounds and admissions to the hospital (CI 95% = 1:05:29 h to 1:25:20 h). In comparison, a physician of a public hospital spent 1:39:29 hours (CI 95% = 1:27:56 h to 1:51:01 h). This result implies a significant difference depending on ownership (chi^2 ^= 24.32, df = 2, p < .001).

The daily duration of direct patient care (including, for example clinical examinations of patients) does not differ significantly from one hospital to the other (chi^2 ^= 1.679, df = 2, p = .432). In addition, a significant negative correlation was found between documentation tasks and direct patient contact (r = -.20, p < .01).

### Differences in communication with patients

Results of the non-parametric analysis showed that physicians of public hospitals communicate significantly more with patients than do physicians of the other two types (chi^2 ^= 30.07, df = 2, p < .001). This category includes the sum of the measured times for patient briefing and diagnostic and therapeutic conversations, as well as for psychological and explanatory talks.

### Additional time

The observed physicians differed significantly in time spent on "walking around between tasks" (chi^2 ^= 19.23, df = 2; p < .001). However, work obstacles such as waiting for reports, patients, colleagues, computer problems or searching documents did not vary significantly (chi^2 ^= .278, df = 2, p = .87). During a working day, physicians of public hospitals spent significantly more time on teaching (e.g. medical students) than physicians working at private hospitals (chi^2 ^= 16.06, df = 2; p < .001).

### Number of patients being treated per day

The univariate test showed physicians of private, for-profit hospitals treated more patients per day (M = 17.43, SD = 2.85) than did physicians of public (M = 16.06, SD = 2.43) or private, non-profit hospitals (M = 14.23, SD = 2.59) (chi^2 ^= 59.36, df = 2, p < .001).

## Discussion

The current study is the first to evaluate physicians work efficiency in German general hospitals and its variation depending on type of ownership using real-time recording. We found evidence showing differences in five major areas depending on the type of hospital ownership: daily working hours, time spent on indirect patient care, administrative duties, direct patient contact and number of patients treated per day.

### Daily working time

Our study results show that physicians' actual daily working time was not optimal in any of the hospitals. The currently monitored physicians work up to 20 hours of overtime per week.

In previous studies, physicians and patients have criticized overtime work in medical care, notably because the risk of medical errors increases significantly if physicians work more than nine hours a day or more than 40 hours per week [19,20]. Furthermore, working overtime is reported to aggravate risk of health problems for physicians themselves [21,22]. Nevertheless, this result indicates an improvement compared to former study results that reported up to 80 working hours per week [23-25]. Fewer working hours might reflect changes in the German working-hour law (since 1 January 2007).

Unexpectedly, our study results showed that physicians of private, for-profit hospitals work significantly fewer hours and have a smaller amount of overtime work, but treated more patients than physicians of public or non-profit hospitals. This outcome can be compared to similar results of the German Federal Office of Statistics showing that the bed productivity (number of patients/number of beds) is higher at private, for-profit hospitals than at public or non-profit hospitals [16].

The combination of these parameters is used as an indicator to measure the efficiency of labour in this study. German hospitals are forced nowadays to operate economically and to avoid financial deficits. This leads to the current situation in which physicians treat more patients per time unit to make a profit and to offset losses. With an increase in numbers of patients treated per time unit, compensation (hospital reimbursement by the insurance companies) for the unit providing health care will increase. Since fixed rates for treatments (case-based lump sum) were introduced as payroll units in 1993 [26] and a "flat-rate" pay system in 2003 based on the DRG classification (Diagnosis Related Groups), the incentive to treat patients more economically grew, particularly in private, for-profit hospitals [27].

Finally, our study results showed that physicians working in public hospitals have to do their documentation tasks and administrative work after regular working hours. Taking into consideration managerial approaches and structures of public hospitals in Germany, we were not surprised to find higher average times regarding indirect patient care and administrative duties in these hospitals.

In public hospitals, an autocratic and extremely bureaucratic organizational and managerial structure is often described and could be linked to the occurrence of indirect patient care duties in these institutions [28]. Previous studies have described similar data concerning the administrative demands [29]. Our study results support this finding as well, and lead to the conclusion that physicians working at public hospitals have to work overtime largely because of more intense documentation and administrative duties.

Although physicians working at private hospitals had more patients to treat, they spent less time on administration and documentation and had generally fewer working hours per day, compared to physicians working at public hospitals. This leads to the question as to whether public hospitals have general organizational deficits, which could explain the connection between a high share of documentation duties, longer working hours per day and even a smaller amount of time spent on direct patient contact and care.

Private, for-profit hospital owners pay strict attention to economical considerations [30]. That is why physicians working at these hospitals are forced to treat more patients per day instead of losing their time on paperwork.

### Time spent on indirect and direct patient care

Regardless of hospital ownership type, our study results show that little time is spent on direct patient care. These outcomes have large ramifications on a physician's performance in the medical system, because direct patient care and contact was found to be of major significance for successful treatment [31-35].

The study results showed that inefficient design of working processes, including an increasing number of documentation duties, causes insufficient direct patient care [36,37]. By reducing tasks on indirect patient care (including administrative duties) and increasing medical tasks in favour of direct patient care, substantial progress would be achieved.

One possibility for modifying the daily working routines is to restructure certain non-medical activities. Former study results showed that implementing a computerized physician order entry and an electronic medical record system would be a positive step forward [38,39]. Additionally, developing an automated process to generate printed discharge instructions and prescriptions were publicized to be helpful as well [40].

### Quality of care

Subsequently we asked whether the differences in relative time of treating more patients per day are achieved at the expense of quality. "Quality of care" is a simple term for a vast and complex field of items that is difficult to distinguish and to measure [41]. A key factor of satisfying medical care depends on effective communication between patients and providers. Ineffective communication can lead to inappropriate diagnosis and/or medical treatment.

The findings of our study illustrated that the acceleration and compression of work are associated with reduced interpersonal contacts – especially those between physicians and patients. This communication time is significantly reduced in private, for-profit hospitals compared to public or private, non-profit hospitals.

The quality of patient-doctor communication depends on different factors, such as duration and intensity, as well as active and passive communication behaviour. Different quality studies have shown that many patients complain about too-short and insufficient conversations [42,43]. Patients feel that they do not get a chance either to describe their personal medical condition completely or to be informed well enough about further procedures. Studies pointed out that a lack of doctor-patient communication often leads to patient dissatisfaction and can cause medical misdiagnoses [44]. As a result, problematic medical errors occur all too frequently [45].

In line with the research pool on this topic, it has to be stated that there are no homogeneous results on "quality of care" so far [46]. Many studies across the health sector have investigated the claim of reduced health care provided by private, for-profit health systems [47-51]. Further results showed that private hospitals, although expected to offer a higher quality of service, fulfilled patients' expectations less than public hospitals [52]. In contrast, there are numerous studies demonstrating that no differences can be found regarding the quality between non-profit and for-profit hospitals, in particular on two indicators, mortality and explicit process [53,54]. Given that our data reflect only one component of the concept "quality of care", other studies must be carried out to be able to comment on other facets of the quality of care.

### Limitations

At this point, it is important to note that our study has some limitations in generalizing the results. The data compiled are not meant to reflect the total population of physicians, nor can we make general statements about all physicians' working flows based upon this limited data set. Although physicians of different medical services and ownerships were included in the study, it is difficult to determine if they are representative, since these physicians were concentrated in only one single geographical area.

Despite these limitations, the results of the study provide significant insight into differences between hospital ownership types regarding physicians' work flow. Considering the limitations, it is highly recommended that further research studies on this subject be conducted. These studies may also take into account other variables that were not included in the current study.

## Conclusion

In summary, the present study points out that type of hospital ownership is a potential factor for variation in physicians' working activities. However, based on our findings, it is not possible to generally state that working activities are performed more efficiently or that quality of care is better with or without a more pronounced commercial focus. But it should be noted that these study results can stimulate an overall improvement of health care services in Germany, not only in the public sector but in private hospitals as well. By using professional, organizational and structural resources more rationally und effectively in German hospitals, the current health care situation could be improved, as considered to be necessary.

## Competing interests

The authors declare that they have no competing interests.

## Authors' contributions

SM and DAG conceived and designed the study. SM managed the data assessment. SM analysed the data. SM wrote the manuscript. SM, CS, KV, DQ, NS, TW, BFK and DAG interpreted the data and contributed substantially to its revision.

## Supplementary Material

Additional file 1 **Table 4. Job task distribution in three ownership categories: Mean ranks (Kruskal-Wallis)**. Table exceeding one A4 page in width. Click here for file
