# Supplementary material for: Does type of hospital ownership influence physicians' daily work schedules? An observational real-time study in German hospital departments
Source: Hum Resour Health. 2009 May 27;7:41. doi: 10.1186/1478-4491-7-41 (PMC2692979; doi:10.1186/1478-4491-7-41)
Supplement: Additional file 1 — Table 4. Job task distribution in three ownership categories: Mean ranks (Kruskal-Wallis). Table exceeding one A4 page in width. [file 1478-4491-7-41-S1.doc]

### Table 4. Job task distribution in three ownership categories: mean ranks (Kruskal-Wallis)

| **Job task category** | **Private, for profit** | | **Public** | | **Private, non-profit** | | **chi²**  (df = 2) |
| --- | --- | --- | --- | --- | --- | --- | --- |
|  | Mean (hh:mm:ss) | CI 95%  (hh:mm:ss) | Mean (hh:mm:ss) | CI 95%  (hh:mm:ss) | Mean (hh:mm:ss) | CI 95%  (hh:mm:ss) |
| Meetings and internal communication | 02:35:21 | 02:23:08 to 02:47:34 | 02:39:02 | 02:29:51 to 02:48:14 | 02:43:41 | 02:34:59 to 02:52:23 | 1.588 |
| Administrative tasks | 01:44:27 | 01:35:45 to 01:53:09 | 01:52:00 | 01:42:48 to 02:01:12 | 01:31:56 | 01:23:32 to 01:40:20 | 7.874* |
| Admission to hospital/  Ward rounds | 01:15:25 | 01:05:29 to 01:25:20 | 01:39:29 | 01:27:56 to 01:51:01 | 01:01:30 | 00:52:36 to 01:10:25 | 24.326*** |
| Indirect patient care | 00:45:49 | 00:39:09 to 00:52:28 | 00:44:56 | 00:36:37 to 00:53:15 | 01:08:02 | 00:58:44 to 01:17:20 | 16.956*** |
| Direct patient care | 00:30:24 | 00:24:33 to 00:36:15 | 00:32:25 | 00:27:12 to 00:37:38 | 00:32:56 | 00:26:58 to 00:38:54 | 1.679 |
| Communication with patients | 00:21:24 | 00:18:34 to 00:24:15 | 00:36:03 | 00:31:17 to 00:40:48 | 00:23:29 | 00:20:01 to 00:26:57 | 30.076*** |
| Resting periods | 00:36:02 | 00:32:22 to 00:39:42 | 00:22:34 | 00:19:47 to 00:25:21 | 00:27:32 | 00:24:21 to 00:30:44 | 28.265*** |
| Walking | 00:34:18 | 00:32:17 to 00:36:19 | 00:41:11 | 00:38:53 to 00:43:29 | 00:40:14 | 00:37:44 to 00:42:44 | 19.237*** |
| Work obstacles | 00:09:07 | 00:07:42 to 00:10:32 | 00:09:08 | 00:07:50 to 00:10:26 | 00:08:54 | 00:07:42 to 00:10:06 | .278 |
| Teaching | 00:00:40 | 00:00:13 to 00:01:08 | 00:07:56 | 00:03:44 to 00:12:08 | 00:02:17 | 00:01:15 to 00:03:18 | 16.056*** |
| Miscellaneous | 00:23:53 | 00:18:20 to 00:29:25 | 00:18:38 | 00:14:39 to 00:22:36 | 00:13:03 | 00:11:09 to 00:14:57 | 5.48* |

*p<.05; **p<.01; ***p<.001; df = degree of freedom
